# Graph measures in task-based fMRI: Functional integration during read-out of visual and auditory information

**DOI:** 10.1371/journal.pone.0207119

**Published:** 2018-11-15

**Authors:** Laura Quante, Daniel S. Kluger, Paul C. Bürkner, Matthias Ekman, Ricarda I. Schubotz

**Affiliations:** 1 Department of Psychology, University of Münster, Münster, Germany; 2 Otto-Creutzfeldt-Center for Cognitive and Behavioral Neuroscience, University of Münster, Münster, Germany; 3 Donders Institute for Brain, Cognition and Behaviour, Radboud University Nijmegen, Nijmegen, Netherlands; 4 Department of Neurology, University Hospital Cologne, Cologne, Germany; South-Central University for Nationalities, CHINA

## Abstract

This study investigated how attending to auditory and visual information systematically changes graph theoretical measures of integration and functional connectivity between three network modules: auditory, visual, and a joint task core. Functional MRI BOLD activity was recorded while healthy volunteers attended to colour and/or pitch information presented within an audiovisual stimulus sequence. Network nodes and modules were based on peak voxels of BOLD contrasts, including colour and pitch sensitive brain regions as well as the dorsal attention network. Network edges represented correlations between nodes’ activity and were computed separately for each condition. Connection strength was increased between the task and the visual module when participants attended to colour, and between the task and the auditory module when they attended to pitch. Moreover, several nodal graph measures showed consistent changes to attentional modulation in form of stronger integration of sensory regions in response to attention. Together, these findings corroborate dynamical adjustments of both modality-specific and modality-independent functional brain networks in response to task demands and their representation in graph theoretical measures.

## Introduction

Integration and segregation in global brain communication are necessary prerequisites for complex behavior [1–3]. While segregation means that distributed areas work independently from one another and serve specialised functions, integration denotes a global coordinative coupling of functionally distinct brain regions.

Using network analysis and graph theoretical measures, the human brain has been shown to be organised in functionally specialised modules [4–8] and a small number of highly connected and topologically central brain regions, the *connective core* (also called *rich club*; [9–12]). The core’s connectivity profile makes it the ideal structure to integrate information from different brain regions. Following Shanahan’s *connective core hypothesis* [13], this central module enables and guides communication between all other brain regions. Parallel computation and competition between brain regions–as coordinated by the connective core—result in the formation of dynamic coalitions of specific brain regions, which in turn determine behavior. Such coalitions are formed in a serial manner, corresponding to a sequential shift between states of integration and segregation [3, 13–14].

Empirical studies have indeed shown that the level of integration within the brain changes depending on task demands [15–18]. Cohen and D’Esposito, for example, found that a working memory task was accompanied by greater integrative communication within the brain when compared to a simple motor task [18]. In addition, Cole and colleagues [19] demonstrated that the fronto-parietal network shows flexible functional connectivity depending on task demands, supporting the idea of a connective core dynamically orchestrating brain processes. Further support for this idea was provided by Ekman and colleagues [20]: In their study, participants prepared for a colour/motion discrimination task. During preparation, colour regions showed higher integration with core regions when colour discrimination was prepared, and reduced integration when motion discrimination was prepared (vice versa for motion regions). In other words, connections between core and periphery dynamically and systematically changed depending on the task to prepare.

In the present study, we investigated changes in core-periphery interaction when participants attended to auditory vs. visual information. Participants were required to attend to colour, pitch, or both colour and pitch information in an audiovisual stimulus sequence. To ensure modality-specific attentional engagement, subjects were asked to not only attend to but read out visual and auditory information to perform a visual search task at the end of each sequence. Importantly, when they interpreted colour, pitch or both colour and pitch as requested, this visual search was facilitated, and performance was enhanced. Note that we defined the joint task network as "core" whereas visual and auditory areas were defined as "periphery". Functionally, the joint task network was specific to the present paradigm and taken to reflect the translation of pitch and/or colour information into the spatial domain for the subsequent search task. Based on Shanahan [13] and in line with Ekman and colleagues [20], we hypothesised temporarily stronger links between task core and task-relevant sensory areas. Specifically, we expected increased functional connectivity between the core and the visual module when participants attended to visual stimuli, and between core and the auditory module when participants attended to auditory stimuli. In either case, there should be stronger integration of the task-specific regions within the network. We assessed integration in terms of functional connectivity and different graph theoretical measures.

## Materials and methods

### Participants

Twenty-eight right-handed volunteers (18–29 years, mean 23.7 ± 2.83 SD years old, 7 male) with normal or corrected-to-normal vision participated in the experiment after giving written informed consent. None of them reported a history of medical, neurological or psychiatric disorders or substance abuse. Participants were compensated with course credit or payment. Two additional participants were excluded because of drop out and heavy leg movements during the fMRI session. The study protocol was conducted in accordance with ethical standards of the Declaration of Helsinki and approved by the local ethics committee of the University of Münster.

### Task

**Fig 1:**
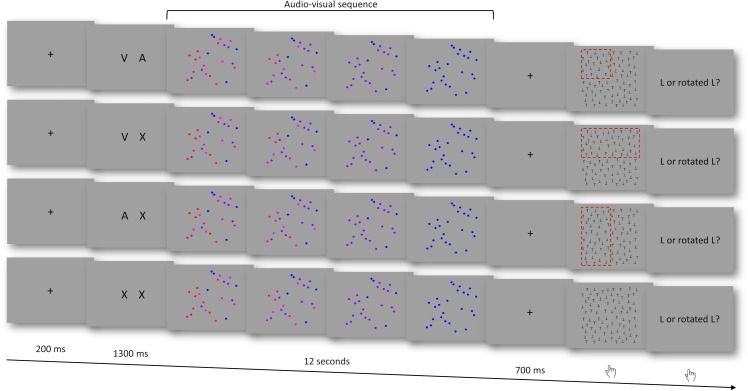
Illustration of the trial design for the four experimental conditions. The area framed in red represents the display location that could be predicted by the participant based on information provided by the audio-visual sequence (note that during the real presentation, no red frame was visible). Functional connectivity analysis was based on data recorded during the entire audio-visual sequence (12 s).

Participants exploited auditory and visual information from a 12-second stimulus sequence to subsequently predict the location of a target in a visual search display (Fig 1). Every trial started with a fixation cross (200 ms) followed by a cue (1300 ms). The deterministic cue indicated which source of information was predictive of the target location and therefore allowed participants to focus on auditory information (A) and/or visual information (V). The cues consisted of two letters (*VA*, *VX*, *AX*, or *XX*) and instructed participants to read out both visual and auditory information (VA), only auditory information (AX), only visual information (VX), or that neither modality was informative (XX). If indicated by the cue, visual information (colour) predicted that the target would appear in the upper or lower half of the search display (e.g., red–upper half, blue–lower half) and auditory information (pitch) indicated that the target would appear in the right or left half of the search display (e.g., high–right half, low–left half). Stimulus-target associations (i.e., red/blue colour–upper/lower half and low/high pitch–right/left side) were balanced across participants. Importantly, in case of condition VA, participants could restrict their visual search to one quarter of the search display, in condition VX and AX to one half. In condition XX, visual search could not be spatially restricted.

The end of the audiovisual sequence was followed by a fixation cross (200 ms) and a blank screen (500 ms) before the visual search display appeared. When the search display appeared, participants were asked to respond with their right index finger to indicate when they detected the target. Next, the response display showed up and participants indicated whether the target was a regular letter “L” (left middle finger) or rotated (left index finger). After the second button press, the next trial started.

Every participant was presented 48 VA trials, 24 VX trials, 24 AX trials, and 48 XX trials in pseudo-randomised order, with overall balanced transition probabilities.

### Stimuli

#### Audio-visual sequences

Participants were presented sequences of 11 audio-visual stimuli which consisted of a visual pattern and a piano-like chord played simultaneously. The visual pattern was a random arrangement of 25 non-overlapping coloured dots, with a dot diameter of 0.3° of visual angle. The dot cloud covered a square area of about 7° of visual angle and was centered on the screen. Each dot was coloured in one of the following five colours: red (RGB values: 255, 0, 0), blue (0, 0, 255), or intermediate mixtures thereof (255, 0, 128; 255, 0, 255; 128, 0, 255). The background was grey (160, 160, 160). Individual stimuli were created with *MATLAB* (The MathWorks, Inc., Natick, Massachusetts, USA) and compiled to videos with *Windows Movie Maker* (Microsoft Cooperation). In each video, stimuli 1 to 10 were presented for 1250 ms, and the last stimulus was presented for 2000 ms. There was a crossfading period of 250 ms, where participants were presented a merged image of two consecutive stimuli. This created a smooth transition from one stimulus to another, resulting in a video duration of 12 seconds. Each video started with a standard stimulus, consisting of a balanced mixture of coloured dots (5 dots per colour). Over the course of the next 10 stimuli presented in each trial, dots changed their colour incrementally until every dot had the same colour (either red or blue). We generated eight different standard stimuli and three types of stepwise changes, leading to 2 final states (red, blue) x 8 standard stimuli x 3 sequence types = 48 different videos.

Auditory stimuli were constructed using synthetic string samples from the *EastWest Colossus sound library* (Native Instruments, Berlin, Germany). Trials were made up of ten successive variations of a five-note *standard* chord (C^maj9^, c–d–e–g–b): Starting with the chord split over five octaves (one note per octave, lasting 1000 ms), notes were individually transposed to the next octave in pseudorandom order every 1000 ms. Following a transposition, the respective note resonated for another 250 ms, creating a smooth transition between chord variations. This way, all notes were gradually shifted upwards or downwards over the course of an auditory sequence until the final chord (lasting 2000 ms) exclusively comprised notes from either the highest or the lowest octave. All notes and corresponding frequencies across octaves are shown in Table 1. As for the visual sequences, we generated 48 different auditory sequences.

**Table 1:** Notes and corresponding frequencies in Hz across octaves.

	Octave					
Note		2	3	4	5	6
	c	65.41	130.81	261.63	523.25	1046.50
	d	73.42	146.83	293.66	587.33	1174.66
	e	82.41	164.81	329.63	659.25	1318.51
	g	98.00	196.00	392.00	783.99	1567.98
	b	123.47	246.94	493.88	987.77	1975.53

For every participant, auditory and visual sequences were randomly paired to build 12 audio-visual sequences of each type (as classified by the final stimulus: blue-high, blue-low, red-high and red-low). Every audio-visual sequence was presented three times per participant: once within condition VA, once within condition XX and once within conditions VX or AX.

#### Visual search display

The visual search display consisted of 80 black letters “T” (Calibri font, 0.4 x 0.2° of visual angle), 40 of which were presented upright and the other 40 upside-down, on a grey background (160, 160, 160). Letters were randomly distributed in a square area of 8.7° of visual angle. For each trial, one letter “T” was replaced by the target (L or rotated L).

### Procedure

The experiment was programmed and run using *Presentation* software (Neurobehavioral Systems, San Francisco, CA, USA).

Participants completed a training session on the first day. The training session comprised 12 trials to learn the association between colour (red and blue) and display location (upper and lower half, respectively), 12 trials to learn the association between pitch (high and low) and display location (right and left half, respectively), as well as 24 trials to learn the association between the combination of colour and pitch and the corresponding display quarter. Afterwards, participants performed 48 training trials of the actual paradigm, i.e. using colour and pitch information to prepare for the upcoming target location (condition VA).

On the second day, the experiment was carried out in the fMRI scanner. Before the experiment, an echo planar imaging sequence was turned on and participants manually adjusted the sound volume. The scanner session was followed by a questionnaire evaluating the participants’ behavior during the session (e.g., strategies, concentration, and difficulties performing the task).

### Data acquisition

Whole-brain images were collected on a 3T Siemens Magnetom Prisma MR tomograph (Siemens, Erlangen, Germany) using a 20-channel head coil. Functional images were acquired using a gradient T2*-weighted single-shot gradient-echo planar sequence sensitive to BOLD contrast (64 × 64 data acquisition matrix, 192 mm field of view, 90° flip angle, repetition time = 2000 ms, echo time = 30 ms). Each volume consisted of 33 adjacent axial slices with a slice thickness of 3 mm and a gap of 1 mm, resulting in a voxel size of 3 × 3 × 4 mm^3^. Images were acquired in interleaved order parallel to the AC-PC line to provide a whole-brain coverage. Structural data were acquired for each participant using a standard Siemens 3-D T1-weighted MPRAGE sequence for detailed reconstruction of anatomy with isotropic voxels (1 × 1 × 1 mm^3^) in a 256-mm field of view (256 × 256 matrix, 192 slices, repetition time = 2130 ms, echo time = 2.28 ms). Participants’ hands were placed on four-button response boxes. Index and middle fingers were placed on the response buttons. To minimise head motion, the head was tightly fixated with cushions, and earplugs were provided to attenuate scanner noise. Auditory stimuli were presented via headphones (MR confon, Magdeburg, Germany). Visual stimuli were projected on a screen positioned behind the participant’s head by a video projector (JVC, Bad Vilbel, Germany). Participants viewed the screen by a 45° mirror, which was fixated on the top of the head coil and adjusted for each participant to provide a good view of the entire screen.

### Behavioral data analysis

Behavioral performance was assessed via error rates and reaction times of correctly answered trials. To test for differences between conditions, pairwise *t*-tests were conducted using the statistic software package *R* (R Foundation for Statistical Computing, Vienna, Austria). Results of the paired *t*-tests were corrected for multiple comparisons at *p* < .05 using Bonferroni correction.

### fMRI data analysis

#### fMRI data preprocessing

Brain image preprocessing and basic statistical analyses were conducted using *SPM12* (Wellcome Trust Centre for Neuroimaging, London, United Kingdom). Functional images were realigned based on three rotation and three translation parameters, slice time corrected, and co-registered to the structural scan. Structural scans were segmented into grey matter, white matter and cerebrospinal fluid. Structural and functional scans were normalised to the Montreal Neuroimaging Institute (MNI) template. Functional images were high-pass filtered (128 s period cutoff) and spatially smoothed with an 8 mm FWHM Gaussian kernel. For connectivity analyses, we additionally performed linear detrending and despiking, and regressed out the six realignment parameters, their temporal derivatives, and the first five PCA components for both white matter and ventricle voxels from the unsmoothed preprocessed functional images using the *CONN* toolbox [21]. For confound removal, the toolbox uses the aCompCor strategy [22].

#### Design specifications

Event-related BOLD responses were estimated using a general linear model approach. The model comprised a total of 15 regressors plus intercept. Regressors of interest represented the four main conditions (VA, VX, AX, and XX), each modeled with an event duration of 12 seconds and convolved with the canonical hemodynamic response function. As regressors of nuisance, we included right and left button presses, cue presentation, visual search and area restriction of the search (as a parametric effect with levels -1, 0, and 1 corresponding to full display, half of the display, and quarter of the display, respectively), as well as the six realignment parameters. The first four regressors of nuisance were convolved with the canonical hemodynamic response function, with the first three being defined as actual events and with visual search being modeled with an event duration corresponding to the reaction time of that trial. Four contrasts were generated for each participant: VX>AX, VA>XX, VX>XX, and AX>XX. For each voxel, resulting contrast weights entered one-sample *t*-tests. General task activations (i.e., attention to visual stimuli, auditory stimuli, or both) were assessed by the conjunction VA>XX ∩ VX>XX ∩ AX>XX. To correct for multiple comparisons, false discovery rate (FDR) correction was used (*p* < .05).

#### Network construction

Nodes represented contrast peak voxels, and edges were defined as correlations between confound-corrected BOLD time series (*BOLD series* hereafter). Sixteen peak voxels were chosen from contrast VA>XX ∩ VX>XX ∩ AX>XX, and six peak voxels each from contrasts VX>AX and AX>VX, resulting in a total of 28 nodes. Every node was created by surrounding the corresponding peak voxel with a 6-mm-radius sphere using *AFNI*’s *3dUndump* and used to average, for every participant, BOLD series of voxels within a sphere (n_voxels_ = 33; see [23]). This approach aims to get reliable time series averages while simultaneously retaining a certain degree of functional node specificity.

Averaged BOLD series were used to calculate Pearson correlations between all pairs of nodes. Correlation coefficients were Fisher’s Z transformed. To generate condition-specific correlation matrices, BOLD series were restricted to relevant volumes from the audio-visual sequences (six TRs per trial) of correctly answered trials, shifted by six seconds to account for the hemodynamic lag. In case of conditions VA and XX, 24 correctly answered trials were randomly chosen to balance the number of trials between conditions (minimum number of trials per subject and condition was 22). Accordingly, every correlation coefficient was based on at least 132 data points.

Matrix construction was done by using customised Python code (Python Software Foundation), utilizing the packages *NumPy* and *NiBabel*, and resulted in one 28 x 28 matrix per participant and condition (112 matrices in total). Connection strength was represented by positive and negative correlation coefficients, whereas negative correlations were set to zero for graph measure calculation (average percentage of negative correlations = 26.6%, range = 6.9–49.7%). MATLAB and the *Brain Connectivity Toolbox* [24] were used to compute connection strength and weighted graph measures. Nodes were grouped into three modules based on contrasts (task module: VA>XX ∩ VX>XX ∩ AX>XX, visual module: VX>AX, and auditory module: AX>VX). The joint task network represented the core of all brain areas involved in task performance.

#### Network analysis

To measure integration and core-periphery interaction, we analyzed connection strengths within and between modules and nodal graph measures by fitting generalised linear multilevel models (GLMMs, [25]). These models include fixed and random effects and thereby account for intra-individual homogeneity and inter-individual heterogeneity [26]. The R package *brms* [27–28], an interface to the programming language *Stan* ([29]; http://mc-stan.org/), was used to estimate the GLMMs in a Bayesian framework [30–31]. We used the default priors of brms, which are chosen to be only weakly informative, thus having only negligible impact on the obtained estimates [27]. The posterior distribution over model parameters was estimated by means of a Markov Chain Monte Carlo procedure and the NUTS sampling algorithm [32] was used to draw samples (two independent chains with 2000 iterations each, of which the first 1000 were used as warm-up, leaving a total of 2000 posterior samples). The mean of the posterior distribution and a credible interval (usually a two-sided 95% credible interval; 95% CI) were used to summarise each model parameter. A 95% CI can be interpreted in the way that a given parameter lies within this interval with 95% probability. If desired, a parameter can be interpreted as significantly different from zero (on a 5% level) if the corresponding 95% CI does not contain zero. In all models, effect coding was used, which means that group means are contrasted with the grand mean by setting a reference category of every independent variable to -1. Because it cannot be assumed that graph measures are normally distributed [33], we chose response distributions that best fit the observed data. Convergence of all Bayesian GLMMs was assessed via the effective sample size for each parameter and the Gelman-Rubin statistic Rhat [34].

To quantify effects of connection type (i.e. within-module and between-modules connections), and attentional modulation on connection strength, we analyzed the data by fitting a GLMM with fixed effects of *visual read-out* (VR; yes, no), *auditory read-out* (AR; yes, no) and *connection type* (CT; visual-visual, auditory-auditory, core-core, visual-auditory, visual-core, auditory-core) and random effects for participant and node. The model specification in R formula (brms) was: Connection Strength ~ VR * AR * CT + (VR * AR | participant) + (VR * AR | node). We chose a skewed normal distribution to describe the distribution of the dependent variable, because the distribution of correlations was right-skewed. For effect coding, the following categories were chosen as reference (i.e., coded with -1): connection type = core-core, visual read-out = no, and auditory read-out = no.

In addition to connection strength, we calculated the following seven weighted graph measures for every node to quantify topological features of the network: betweenness centrality, characteristic path length, clustering coefficient, core closeness, nodal efficiency, participation coefficient, and strength (see S1 Table for detailed information).

For each graph measure, we fitted a GLMM with fixed effects of *visual read-out* (VR; yes, no), *auditory read-out* (AR; yes, no) and *module* (MOD; visual, auditory, core) and random effects for participant and node. The model specification in R formula (brms) was: Graph Measure ~ VR * AR * MOD + (VR * AR | participant) + (VR * AR | node). For effect coding, the following categories were chosen as reference (i.e., coded with -1): module = core, visual read-out = no, and auditory read-out = no.

## Results

### Behavioral results

**Fig 2:**
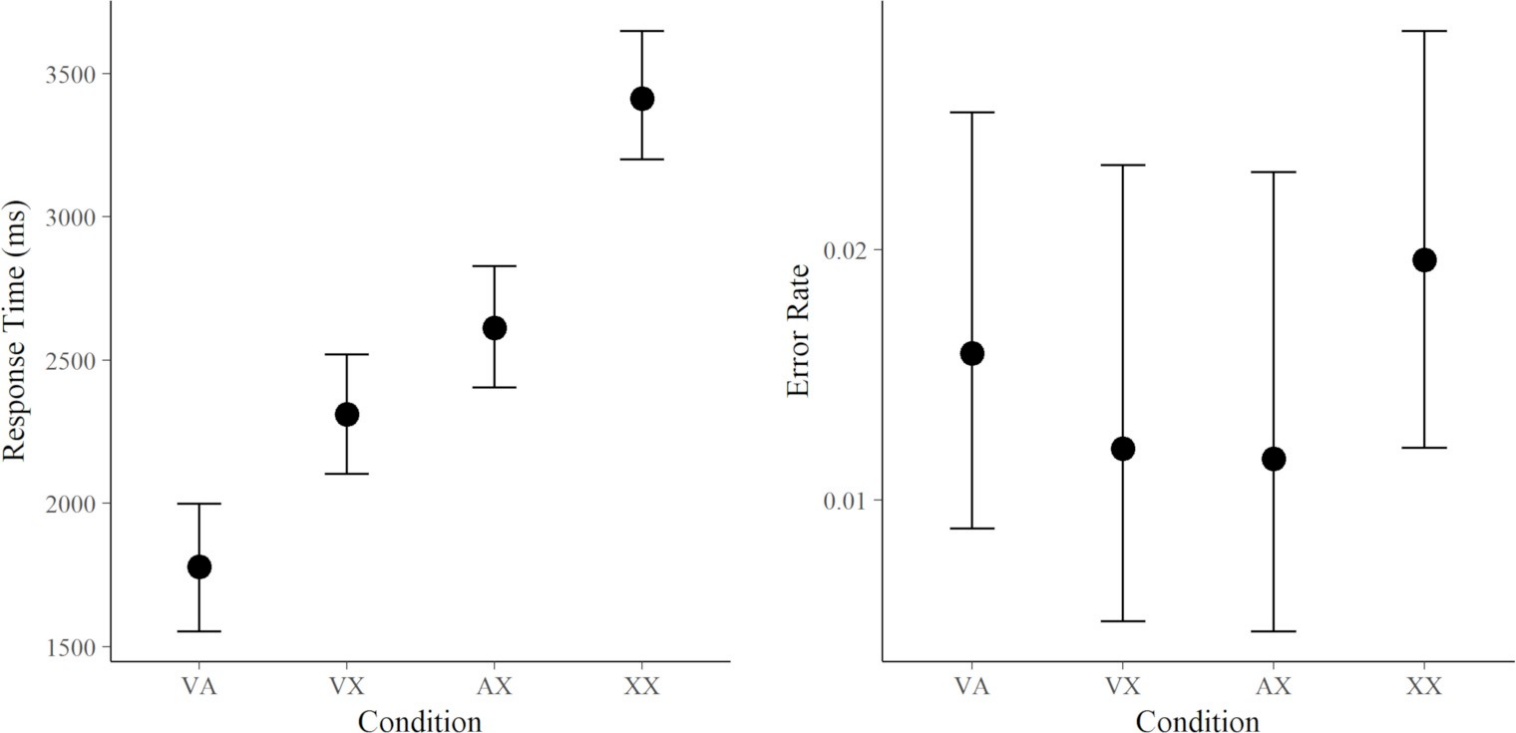
Mean reaction times and error rates for each condition of the fMRI experiment. Error bars represent confidence limits (95%). VA = visual and auditory read-out, VX = only visual read-out, AX = only auditory read-out, XX = control condition (no read-out). Reaction times show that visual search was not informed (restricted) in the XX condition, more restricted in the VX and the AX conditions, and most restricted in the VA condition.

Performance during training and fMRI session was assessed through error rates and reaction times on correctly answered trails. The average error rate of the fourth part of the training, which corresponded to condition VA, was 3.27% (± 3.42 SD) and the average reaction time was 1659.80 ms (± 527.91 SD), both demonstrating participants’ ability to perform the task correctly. The average error rate of the fMRI session was 1.61% (± 1.52 SD; range 0–4.86%) and did not differ significantly between conditions (*p* > .05 for all pairwise *t*-tests). The average reaction time was 2507.48 ms (± 613.93 SD) and differed significantly between conditions (*p* ≤ .001 for all pairwise *t*-tests), indicating that participants used the provided visual and auditory information to predict the target location. See Fig 2 and Table 2 for average reaction times and error rates per condition.

**Table 2:** Mean and standard deviation of reaction times and error rates during the fMRI session. Condition abbreviation see text and legend of Fig 2.

	VA	VX	AX	XX
Reaction time (ms)	1551 (549)	2211 (735)	2657 (917)	3611 (749)
Error rate (%)	1.64 (2.49)	1.19 (2.23)	1.19 (2.50)	2.01 (2.57)

### fMRI results

#### Contrasts

In order to derive nodes for our network analysis, we focused on the following three contrasts (see Table 3): Group-level activations related to visual read-out (VX > AX) were found bilaterally in the occipital cortex and fusiform gyrus (BA 18/19/37) and in the superior parietal lobule (BA 7). We used the three most strongly activated voxels of the biggest cluster within the left and right hemisphere as nodes. The reverse contrast (AX > VX), representing auditory read-out, revealed significant bilateral activations in the superior temporal gyrus (BA 22). As nodes we used the three peak voxels of the left cluster, the peak voxel of the right cluster and two additional right hemisphere voxels, which corresponded to the significant left hemisphere voxels. This procedure ensured equal numbers of nodes within the visual and auditory modules. The conjunction of visual and/or auditory read-out relative to the passive condition (VA>XX ∩ VX>XX ∩ AX>XX) yielded significant bilateral activations in the superior and inferior parietal lobule along the intraparietal sulcus (BA 7/39/40), in the dorsal premotor cortex and inferior frontal junction (BA 6/44), and in lobule VI of the cerebellum. Sixteen voxels were chosen from this contrast to represent nodes of the task module.

**Table 3:** Peak voxel and local maxima.

Area		Peak voxels and local maxima					Cluster size		p-value		Node
		MNI coordinates			z-value	p-value					
		x	y	z							
**Visual (VX > AX)**												
Occipital cortex and fusiform gyrus (BA 18/19/37)	L	-27	-88	2	5.79	< .001	1287	< .001 (< .001)		yes	
		-42	-70	-10	5.44	< .001				yes	
		-33	-76	-13	5.37	< .001				yes	
Occipital cortex and fusiform gyrus (BA 18/19/37), superior parietal lobule (BA 7)	R	33	-82	5	5.78	< .001	1892	< .001 (< .001)		yes	
		42	-73	-7	5.49	< .001				yes	
		48	-61	-10	5.45	< .001				yes	
		24	-55	56	4.74					no	
Superior parietal lobule (BA 7)	L	-18	-58	68	4.12	.001	221	.004 (.044)		no	
		-27	-55	62	3.97	.002				no	
Thalamus	L	-18	-31	11	3.75	.003	114	.028 (.279)		no	
Prefrontal cortex (BA 10/32)	L	-12	38	-4	3.61	.005	258	.002 (.024)		no	
Putamen	R	36	-7	2	3.59	.005	153	.013 (.140)		no	
**Auditory (AX > VX)**												
Superior temporal gyrus (STG; BA 22/41/42)	L	-54	-37	17	4.64	.022	60	.008 (.011)		yes	
		-65	-34	11	3.48		n.s.			yes	
		-66	-25	5	2.47		n.s.			yes	
	R	66	-34	11	4.62	.022	125	< .001 (.001)		yes	
		54	-34	11	4.28	.022				yes	
		66	-25	5	4.27	.022				yes	
**Task (VA>XX ∩ VX>XX ∩ AX>XX)**												
Inferior and superior parietal lobule (BA 7/39/40)	L	-33	-43	41	5.98	< .001	993	< .001 (< .001)		yes	
		-27	-55	44	5.98	< .001				yes	
		-18	-70	53	5.09	< .001				yes	
Inferior and superior parietal lobule (BA 7/39/40)	R	12	-64	56	4.59	< .001	323	< .001 (.002)		yes	
		30	-55	41	4.50	< .001				yes	
Dorsal premotor cortex and inferior frontal junction (BA 6/44)	L	-45	2	26	5.26	< .001	1011	< .001 (< .001)		yes	
		-57	5	20	5.16	< .001				yes	
		-30	-1	59	4.87	< .001				yes	
		-5	6	57	3.76					yes	
	R	42	5	26	4.94	< .001	431	< .001 (< .001)		yes	
		24	2	59	4.22	.001				yes	
		36	-4	41	4.07	.002				yes	
Cerebellum (lobule VI)	L	-24	-64	-25	4.04	.002	113	.015 (.148)		yes	
		-27	-55	-28	3.98	.002				yes	
		-12	-70	-25	3.46	.009				yes	
	R	30	-64	-25	4.10					yes	

*Note*. Shown are local peaks of significant clusters (n.s. = not significant).

#### Network analysis

Mean, standard deviation and range of all conditions are provided for graph measures and connection strength in S2 Table. All GLMMs converged successfully with an effective sample size of 300 or more for every relevant parameter and Rhat values of less than 1.1.

**Fig 3:**
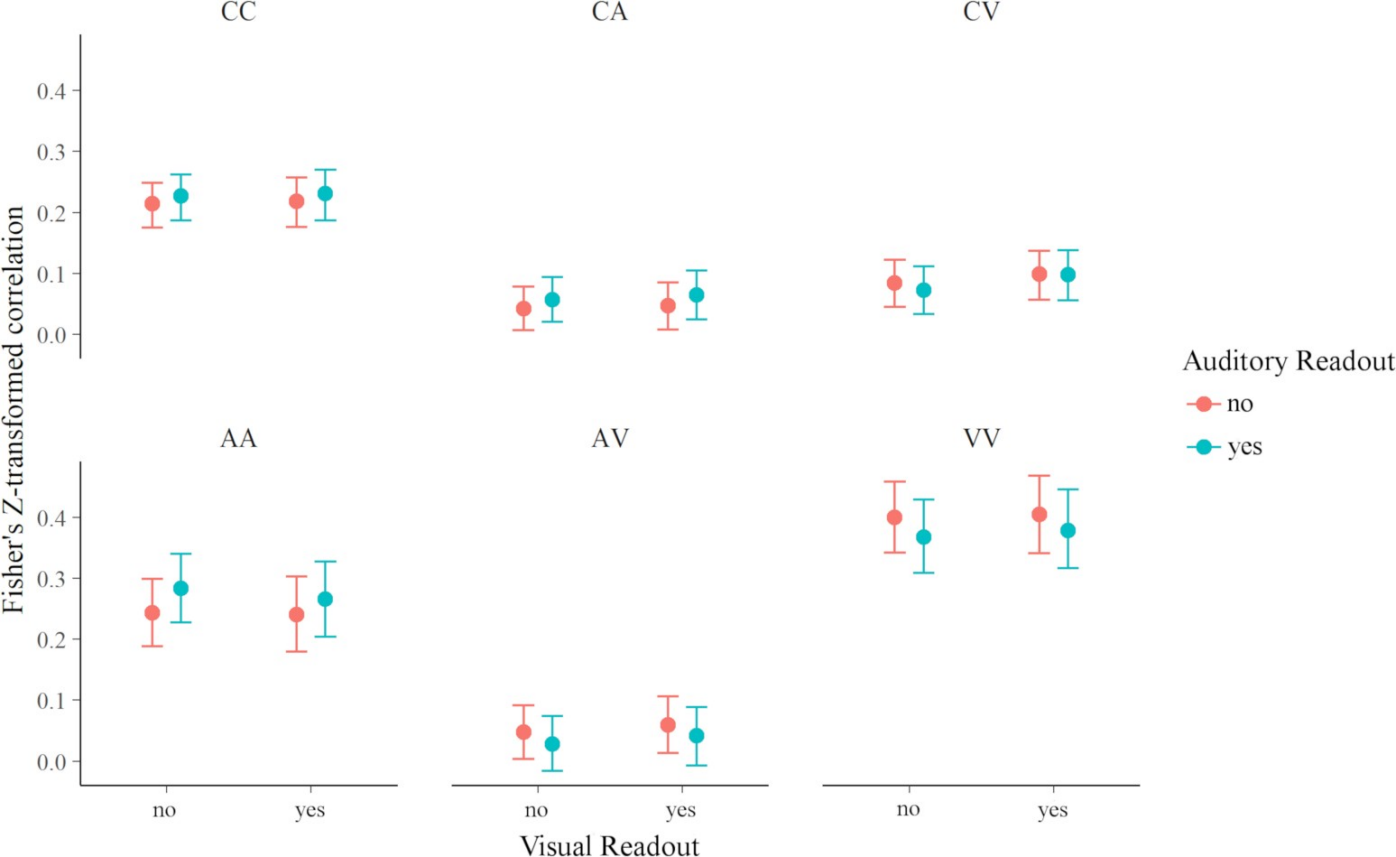
Connection strength for each connection type. Error bars represent 95% credible intervals. CC = connections within the task core, CA = connections between task core and auditory module, CV = connections between task core and visual module, AA = connections within auditory module, AV connections between auditory and visual module, VV = connections within visual module.

**Fig 4:**
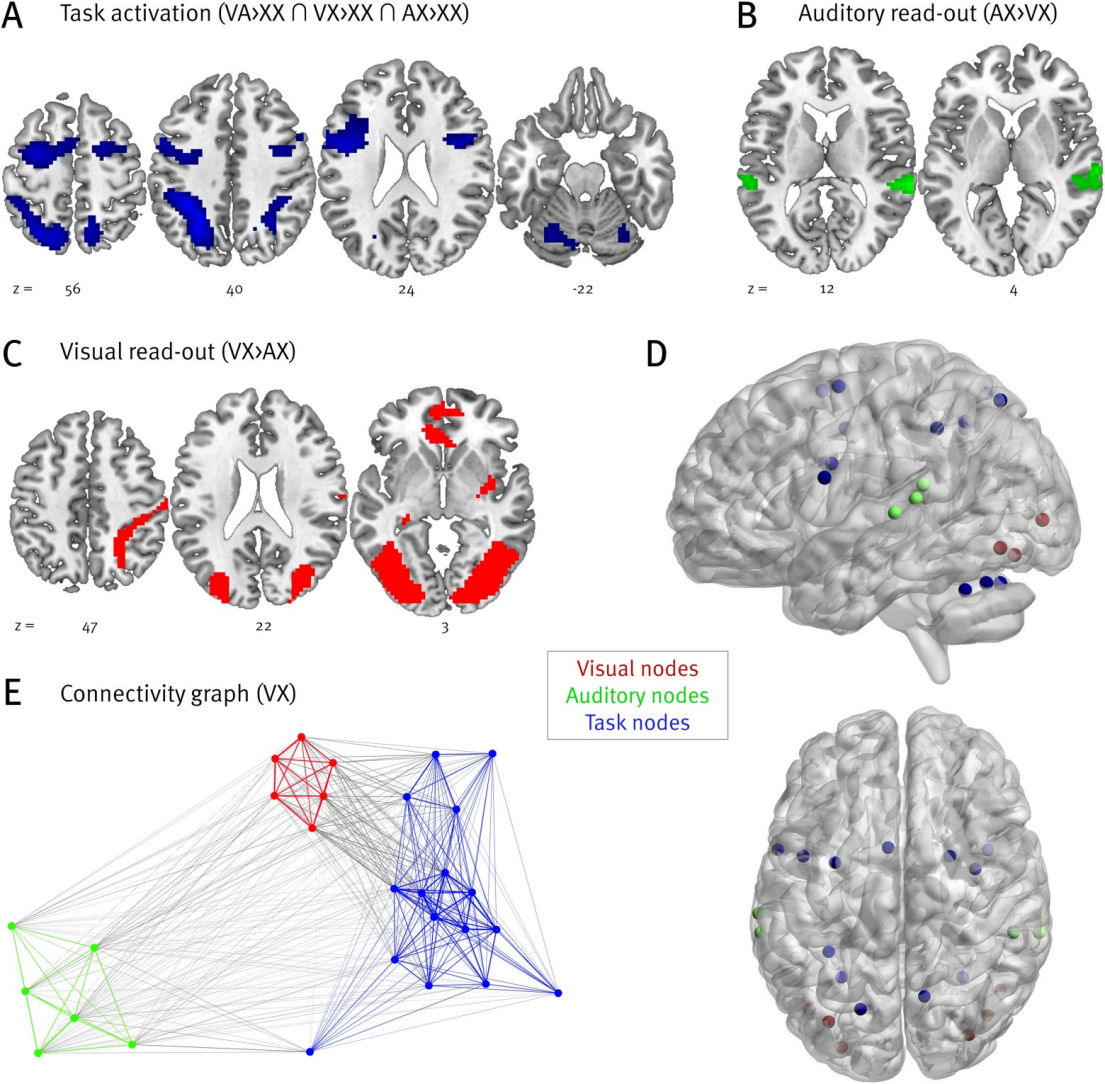
(A)–(C) Significant activations of the three contrasts of interest (FDR corrected, p < .05). (D) Nodes of the network analysis based on contrasts. The figure was generated with BrainNet Viewer [35]. (E) Connectivity graph of condition VX; only positive connections are displayed. A similar module structure was observed for conditions VA, AX, and XX. The figure was generated with Gephi [36] using the ForceAtlas2 algorithm [37]. Arrangement of nodes and edge width are based on connectivity strength.

Connection strength of all connection types differed from the grand mean, with within-module connections being stronger and between-module connections being weaker (see Fig 3 and Table 4). When visual information was read out, connections between the visual and task module were stronger (*b* = 0.007, 95%-CI = [0.003, 0.011]) and connections within the auditory module were weaker (*b* = -0.009, [-0.017, -0.001]; see Fig 4). When auditory information was read out, connections between the auditory and task module (*b* = 0.008, [0.004, 0.011]) and connections within the auditory module were stronger (*b* = 0.015, [0.008, 0.023]), and connections within the visual module (*b* = -0.015, [-0.023, -0.007]) and connections between the visual and auditory module were weaker (*b* = -0.01, [-0.015, -0.005]).

**Table 4:** GLMM for connection strength: Fixed effects.

Population-Level Effect	Estimate	l-95% CI	u-95% CI	
Intercept	0.176	0.141	0.210	*
VR_yes	0.003	-0.003	0.009	
AR_yes	0.001	-0.005	0.007	
CT_TA	-0.122	-0.143	-0.103	*
CT_TV	-0.087	-0.108	-0.067	*
CT_AA	0.082	0.042	0.124	*
CT_AV	-0.131	-0.148	-0.113	*
CT_VV	0.212	0.172	0.254	*
VR_yes * AR_yes	0.000	-0.004	0.005	
VR_yes * CT_TA	0.000	-0.004	0.004	
VR_yes * CT_TV	0.007	0.003	0.011	*
VR_yes * CT_AA	-0.009	-0.017	-0.001	*
VR_yes * CT_AV	0.003	-0.002	0.008	
VR_yes * CT_VV	0.000	-0.007	0.008	
AR_yes * CT_TA	0.008	0.004	0.011	*
AR_yes * CT_TV	-0.004	-0.007	0.000	
AR_yes * CT_AA	0.015	0.008	0.023	*
AR_yes * CT_AV	-0.010	-0.015	-0.005	*
AR_yes * CT_VV	-0.015	-0.023	-0.007	*
VR_yes * AR_yes * CT_TA	0.001	-0.003	0.004	
VR_yes * AR_yes * CT_TV	0.002	-0.001	0.006	
VR_yes * AR_yes * CT_AA	-0.004	-0.011	0.003	
VR_yes * AR_yes * CT_AV	0.000	-0.004	0.005	
VR_yes * AR_yes * CT_VV	0.001	-0.006	0.008	

*Note*. Credible intervals not including zero are marked with *. VR = visual read-out, AR = auditory read-out, CT = connection type with A = auditory, T = task, V = visual.

Table 5 displays credible intervals for the population-level effects of the GLMMs fitted for each graph measure.

**Table 5:** Credible intervals of fixed effects of graph measure GLMMs.

Population-Level Effect	Betweenness centrality	Characteristic path length	Clustering coefficient	Core closeness	Nodal efficiency	Participation coefficient	Strength
Intercept	**[2.88; 3.32]**	**[1.73; 1.88]**	**[-1.94; -1.71]**	**[-4.63; -4.43]**	**[0.157; 0.181]**	**[0.439; 0.505]**	**[1.16; 1.42]**
VR_yes	[-0.10; 0.03]	[-0.04; 0.00]	[-0.01; 0.04]	[-0.00; 0.03]	[0.000; 0.006]	[-0.001; 0.014]	[-0.00; 0.04]
AR_yes	[-0.08; 0.04]	[-0.03; 0.01]	[-0.02; 0.04]	[-0.00; 0.04]	[-0.002; 0.005]	[-0.009; 0.001]	[-0.01; 0.04]
MOD_aud	[-0.35; 0.30]	**[0.06; 0.17]**	**[-0.30; -0.12]**	**[-0.33; -0.15]**	**[-0.028; -0.010]**	**[0.021; 0.105]**	**[-0.40; -0.12]**
MOD_vis	[-0.31; 0.32]	[-0.09; 0.02]	[-0.02; 0.18]	[-0.15; 0.03]	[-0.006; 0.014]	**[0.001; 0.081]**	[-0.05; 0.24]
VR_yes * AR_yes	[-0.09; 0.04]	[-0.02; 0.01]	[-0.03; 0.02]	[-0.02; 0.02]	[-0.002; 0.003]	[-0.005; 0.006]	[-0.02; 0.02]
VR_yes * MOD_aud	[-0.14; 0.04]	[-0.00; 0.02]	[-0.03; 0.01]	[-0.02; 0.01]	[-0.004; 0.000]	[-0.006; 0.006]	[-0.04; 0.01]
VR_yes * MOD_vis	[-0.07; 0.11]	**[-0.02; -0.002]**	[-0.00; 0.04]	**[0.01; 0.03]**	[0.000; 0.003]	[-0.007; 0.006]	[-0.00; 0.04]
AR_yes * MOD_aud	[-0.05; 0.13]	**[-0.03; -0.01]**	**[0.02; 0.06]**	**[0.01; 0.04]**	**[0.001; 0.004]**	[-0.004; 0.007]	**[0.02; 0.07]**
AR_yes * MOD_vis	[-0.15; 0.04]	**[0.02; 0.04]**	**[-0.06; -0.03]**	**[-0.04; -0.02]**	**[-0.005; -0.002]**	[-0.008; 0.003]	**[-0.08; -0.03]**
VR_yes * AR_yes * MOD_aud	[-0.13; 0.05]	[-0.00; 0.02]	[-0.02; 0.02]	[-0.02; 0.01]	[-0.002; 0.001]	[-0.008; 0.004]	[-0.03; 0.01]
VR_yes * AR_yes * MOD_vis	[-0.08; 0.10]	[-0.02; 0.00]	[-0.01; 0.02]	[0.00; 0.03]	[-0.001; 0.002]	[-0.004; 0.008]	[-0.01; 0.04]

*Note*. Credible intervals not including zero are written in bold. VR = visual read-out, AR = auditory read-out, MOD = module with vis = visual, aud = auditory.

Nodes within the auditory module had longer paths to other nodes (*b* = 0.118, 95%-CI [0.061, 0.174]), showed reduced clustering (*b* = -0.208, [-0.298, -0.116]), a greater distance to the core (i.e., task module; *b* = -0.236, [-0.326, -0.146]), decreased efficiency (*b* = -0.019, [-0.028, -0.010]) and decreased strength (*b* = -0.262, [-0.404, -0.117]) as well as higher participation in different modules (*b* = 0.063, [0.021, 0.105]), when compared to the grand mean.

Nodes within the visual module showed higher participation in different modules (*b* = 0.042, [0.001, 0.081]), when compared to the grand mean.

When auditory information was read out, auditory nodes had shorter paths to other nodes (*b* = -0.019, [-0.028, -0.010], showed enhanced clustering (*b* = 0.037, [0.018, 0.055]), a smaller distance to the core (*b* = 0.024, [0.012, 0.036]), and increased efficiency (*b* = 0.002, [0.001, 0.004] as well as increased strength (*b* = 0.046, [0.023, 0.069]). The opposite pattern was observed for visual nodes: They had longer paths to other nodes (*b* = 0.026, [0.017, 0.035]), showed reduced clustering (b = -0.044, [-0.062, -0.026]), a greater distance to the core (*b* = -0.031, [-0.043, -0.019]), as well as decreased efficiency (*b* = -0.004, [-0.005, -0.002]) and decreased strength (*b* = -0.057, [-0.079, -0.034]).

When visual information was read out, visual nodes had shorter paths to other nodes (b = -0.011, [-0.021, -0.002]) and were closer to the core (b = 0.018, [0.005, 0.030]). A summary of network analysis results is provided in Table 6.

**Table 6:** Summary of results from network analysis.

	Auditory read-out				Visual read-out			
**Connection strength**	A–T		↑		V–T		↑	
	A–A		↑		A–A		↓	
	V–V		↓					
	V–A		↓					
**Graph measures**	Auditory nodes		Visual nodes		Auditory nodes		Visual nodes	
	PL	↓	PL	↑			PL	↓
	CL	↑	CL	↓				
	CCL	↑	CCL	↓			CCL	↑
	E	↑	E	↓				
	S	↑	S	↓				

*Note*. V = Visual module, A = Auditory module, T = task module (core); PL = characteristic path length, CL = clustering coefficient, CCL = core closeness, E = nodal efficiency, S = strength.

## Discussion

This study investigated how attending to auditory and visual information systematically changes graph theoretical measures of integration and functional connectivity between three network modules: auditory, visual, and joint task core. Supporting our hypotheses, connection strength was increased between the task and the visual module when participants attended to colour, and between the task and the auditory module when they attended to pitch. Moreover, several nodal graph measures showed consistent changes to attentional modulation in form of stronger integration of sensory regions in response to attention. Together, these findings corroborate dynamical adjustments of both modality-specific and modality-independent functional brain networks in response to task demands and their representation in graph theoretical measures.

In a first step, we analyzed functional segregation, i.e., the specialization of a brain region for a specific function, to determine our network nodes. By computing BOLD contrasts, we established three sets of areas specific for visual read-out, for auditory read-out, and for the common task shared by both. Attention to either visual or auditory information provided by the audiovisual stimulus sequence led to enhanced activity in relevant sensory brain regions. The read-out of colour information led to activations in V2, V4, and inferior-temporal cortex which are all involved in colour processing [38–39]. Accompanying activity in the dorsal visual stream presumably resulted from the spatial arrangement of the colour-changing circles [40–41], but was omitted for node definition. In case of auditory read-out, we found bilateral activity in Heschl’s gyrus (BA 41 and 42) reflecting pitch processing [42–43], and in posterior STG (BA 22) reflecting complex auditory processing, as required for chords and melodies [42, 44–45]. Right hemisphere activations were slightly more pronounced, as often found for music [42, 44–46]. No matter whether participants attended to pitch, colour, or both, the common task was translating pitch and colour information into the spatial domain to prepare for the upcoming visual search. This task elicited bilateral activity in the frontal eye fields/dorsal premotor cortex, superior parietal lobule, and intraparietal sulcus–brain regions known as the dorsal attention network (DAN; [47–50]). The DAN is involved in spatial and non-spatial allocation of attention [51–53] and arbitrary sensorimotor mapping [54–56], both crucial functions for the present task. Notably, DAN activity is not restricted to the visual domain but also found for touch [57–58] and audition [59–61], illustrating the network’s multimodality [62–63]. As for all fronto-parietal networks residing in the association cortex, the DAN is highly and reciprocally interconnected to lower-level primary and secondary sensory cortices [64–66], making it the ideal core among our network modules.

In a second step, we analyzed functional integration, i.e., the coordinative coupling of functionally distinct brain regions. Peak voxels of the calculated BOLD contrasts constituted the centers of our network nodes, and every node was assigned to either the visual, auditory, or task network (core). Note that the definition of our core was not based on graph theoretical measures but theoretical assumptions. We were particularly interested in the interaction between core and sensory modules and the functional integration of individual nodes. Functional connectivity between nodes was measured in terms of (Z-transformed) Pearson correlations between two nodes’ BOLD time series, and weighted graph measures were based on all positive correlations. The graph theoretical analysis revealed that several nodal graph measures were sensitive to attentional modulation. During auditory read-out, auditory nodes were characterised by shorter characteristic paths, increased node strength, increased nodal efficiency as well as increased clustering. These measures can be interpreted as reflecting enhanced network communication and, therefore, stronger integration [24]. Interestingly, the opposite pattern was observed for visual nodes in the same periods, which means that they were less integrated when attention was directed to auditory information. In contrast, when visual information was read out, visual nodes exhibited shorter paths, which again demonstrated enhanced integration during attention. In accordance with our hypotheses, attention to either visual or auditory information led to enhanced integration of corresponding sensory nodes. Visual nodes even showed reduced integration when attention was directed to audition. Although not part of our original hypotheses, the finding of reduced integration of visual nodes during auditory task-relevance replicates the results from an earlier study [20] and demonstrates the network’s ability to flexibly adapt to current task demands. More specifically, both studies reported reduced clustering as well as core closeness for task-irrelevant areas. Conceptually, the clustering coefficient reflects interconnectivity, meaning that a highly clustered network consists of nodes whose neighbours are themselves neighbours. Reduced clustering of visual nodes during auditory read-out–importantly, along with *enhanced* clustering of auditory nodes—thus reflects an exceptionally effective adaptation of network connectivity. Fittingly, reduced core closeness of visual nodes during auditory read-out (again, along with *enhanced* core closeness of auditory nodes) shows that not only within-module connections but also core-periphery interactions were flexibly and efficiently reorganised to fit current task demands. Our results thus further extend previous findings in that enhanced integration measures are not specific to the visual system but also apply to auditory circuits, indicating a modality-independent adaptive process.

To further assess the interactions between sensory modules and task core, we considered effects of visual and auditory attention on within-module and between-modules connection strength by fitting a GLMM. The analysis of between-modules connections revealed a dynamic, task-dependent coupling of our task core and sensory modules. As hypothesised, modality-specific regions were more strongly connected to the task core when the corresponding modality was attended to. Previous studies have shown this separately for the visual [67–71] and the auditory domain [72], whereas our study is the first to demonstrate this effect for both modalities within one experiment and based on combined audiovisual stimuli. Our results suggest that during attention, core-periphery communication is dynamically adjusted to fulfill task demands. While core-periphery communication was equally modulated by attention for both visual and auditory modules, we found reduced connection strength within the module processing the unattended modality as well as increased connection strength within the auditory module in case of auditory read-out. Notably, the latter effect was absent for the visual module during visual read-out. These results are in contrast to studies reporting reduced connection strength within the visual module in a visual task when compared to rest [69] or passive viewing [71]. Thus, further work is needed to clarify differences between visual and auditory within-module modulation.

## Conclusion

Using a graph theoretical analysis approach to task-based fMRI, core-periphery interaction and integration for vision and audition can be demonstrated within one experiment and based on the same stimuli. In response to visual and auditory selective attention, increased functional connectivity between task-relevant sensory regions and the dorsal attention network, and nodal graph measures signify enhanced integration of sensory nodes in response to attention. These findings illustrate the brain’s ability to dynamically adjust network communication to fulfill task-demands. Given that the use of graph theoretical measures in task-based fMRI research is still in its infancy, this study adds to the recently burgeoning evidence that graph measures are valuable for capturing dynamic cognitive processes.

## Supporting information

S1 Table Description, formula, value range, response distribution, and link function of analyzed graph measures. (DOCX)

S2 Table Mean, standard deviation, and range of all conditions for the assessed graph measures and connection strength. (DOCX)
