# Supplementary material for: Graph measures in task-based fMRI: Functional integration during read-out of visual and auditory information
Source: PLoS One. 2018 Nov 15;13(11):e0207119. doi: 10.1371/journal.pone.0207119 (PMC6237351; doi:10.1371/journal.pone.0207119)
Supplement: S1 Table — (DOCX) [file pone.0207119.s001.docx]

**S1 Table.** Description, formula, value range, response distribution and link function of analyzed graph measures.

| Graph measure | Description and formula | Range | Response distribution ^a^ | Link function (GLMM) |
| --- | --- | --- | --- | --- |
| Betweenness centrality ^d^ | Fraction of all shortest paths that contain a given node  $C_{B}\left( i \right)=\sum_{h\neq i,h\neq j,j\neq i} \frac{\rho_{hj}\left( i \right)}{\rho_{hj}}$  $C_{B}\left( i \right)$= betweenness centrality of node *i*  $\rho_{hj}\left( i \right)$ = number of shortest paths between *h* and *j* that pass through *i*  $\rho_{hj}$ = number of shortest paths between *h* an *j* | 0 – 351 ^b^ | negative binomial | log |
| Characteristic path length ^d^ | Average of the shortest paths from a given node to all other nodes  $l\left( i \right)=\frac{1}{N-1}\sum_{i\neq j} l_{ij}$  $l\left( i \right)$= average shortest path length from node *i* to all other nodes  $l_{ij}$ = shortest path length from node *j* to node *i*  *N* = number of nodes in the network | 0 – inf | lognormal | identity |
| Clustering coefficient ^d^ | Proportion of a given node’s neighbors that are neighbors as well  ${Cl}^{w}\left( i \right)=\frac{2}{k_{i}\left( k_{i}-1 \right)}\sum_{j,h} \left( \hat{w}_{ij}\hat{w}_{jh}\hat{w}_{hi} \right)^{1/3}$  ${Cl}^{w}\left( i \right)$ = weighted clustering coefficient of node *i*  $k_{i}$ = total degree of node *i*  $\hat{w}_{ij}$ = scaled edge weight between node *i* and node *j* (relative to the maximum edge weight in the network: $\hat{w}_{ij}\leftarrow w_{ij}/max\left( w \right)$) | 0 – 1 | beta | logit |
| Core closeness ^e^ | Inverse average distance of a given node to all core nodes  ${CCL}_{i\in P}=\frac{1}{\sum_{j\in C} l_{ij}}$  ${CCL}_{i\in P}$ = core closeness of node *i*  $l_{ij}$ = shortest path length from node *i* to node *j*  *P* = periphery  *C* = core | 0 – 1 | lognormal | identity |
| Nodal efficiency ^d^ | Inverse of the characteristic path length; ease of communication between a given node and the rest of the network  $E_{nodal}\left( i \right)=\frac{1}{N-1}\sum_{i\neq j} \frac{1}{l_{ij}}$  $E_{nodal}\left( i \right)$= nodal efficiency of node *i*  $l_{ij}$ = shortest path length from node *j* to node *i*  *N* = number of nodes in the network | 0 – 1 | Gaussian | identity |
| Participation coefficient ^f^ | A given node’s diversity of intermodular connections  $P_{i}=1-\sum_{s=1}^{N_{M}} \left( \frac{\kappa_{is}}{k_{i}} \right)^{2}$  $\kappa_{is}$ = weight of the edges linking node *i* to nodes in module *s*  $k_{i}$ = strength of node *i* | 0 – 1 | skew normal ^c^ | identity |
| Strength ^d^ | Sum of a given node’s edge weights  $s_{i}=\sum_{j\neq i} w_{ij}$  $s_{i}$ = strength of node i  $w_{ij}$ = weight of the edge linking nodes i and j | 0 – inf | lognormal | identity |

^a^ Response distributions were chosen based on data observation

^b^ (N-1)*(N-2)/2 with N = number of nodes

^c^ A skew normal distribution was chosen instead of a beta distribution

^d^ Fornito et al. (2016); ^e^ Ekman et al. (2012); ^f^ Guimerà & Amaral (2005)
