# Supplementary material for: Graph measures in task-based fMRI: Functional integration during read-out of visual and auditory information
Source: PLoS One. 2018 Nov 15;13(11):e0207119. doi: 10.1371/journal.pone.0207119 (PMC6237351; doi:10.1371/journal.pone.0207119)
Supplement: S2 Table — (DOCX) [file pone.0207119.s002.docx]

**S2 Table.** Mean, standard deviation, and range of all conditions for the assessed graph measures and connection strength.

| **Graph Measure** | **Module** | **Condition** | **Mean** | **SD** | **Range** |
| --- | --- | --- | --- | --- | --- |
| Betweenness Centrality | Core | VA | 26.0402 | 6.8729 | 28.2500 |
|  |  | AX | 25.9107 | 6.8007 | 26.2500 |
|  |  | VX | 25.2277 | 6.0950 | 26.0000 |
|  |  | XX | 26.7232 | 6.6240 | 27.1250 |
|  | Auditory | VA | 19.0476 | 8.2590 | 29.6667 |
|  |  | AX | 24.8810 | 8.7968 | 34.6667 |
|  |  | VX | 21.1786 | 8.5714 | 41.0000 |
|  |  | XX | 21.7500 | 8.4374 | 32.0000 |
|  | Visual | VA | 21.2619 | 9.6380 | 38.3333 |
|  |  | AX | 23.0357 | 8.9080 | 33.0000 |
|  |  | VX | 25.5000 | 13.6585 | 65.0000 |
|  |  | XX | 25.9762 | 11.2191 | 58.6667 |
| Clustering Coefficient | Core | VA | 0.1603 | 0.0442 | 0.2047 |
|  |  | AX | 0.1600 | 0.0370 | 0.1555 |
|  |  | VX | 0.1591 | 0.0364 | 0.1318 |
|  |  | XX | 0.1550 | 0.0339 | 0.1365 |
|  | Auditory | VA | 0.1228 | 0.0316 | 0.1356 |
|  |  | AX | 0.1227 | 0.0227 | 0.1076 |
|  |  | VX | 0.1143 | 0.0318 | 0.1277 |
|  |  | XX | 0.1141 | 0.0316 | 0.1321 |
|  | Visual | VA | 0.1522 | 0.0357 | 0.1510 |
|  |  | AX | 0.1433 | 0.0409 | 0.1794 |
|  |  | VX | 0.1610 | 0.0460 | 0.1511 |
|  |  | XX | 0.1534 | 0.0384 | 0.1350 |
| Core Closeness | Core | VA | 0.0158 | 0.0039 | 0.0174 |
|  |  | AX | 0.0157 | 0.0036 | 0.0147 |
|  |  | VX | 0.0151 | 0.0034 | 0.0130 |
|  |  | XX | 0.0149 | 0.0031 | 0.0127 |
|  | Auditory | VA | 0.0092 | 0.0017 | 0.0064 |
|  |  | AX | 0.0091 | 0.0014 | 0.0057 |
|  |  | VX | 0.0086 | 0.0017 | 0.0060 |
|  |  | XX | 0.0084 | 0.0021 | 0.0091 |
|  | Visual | VA | 0.0109 | 0.0025 | 0.0097 |
|  |  | AX | 0.0100 | 0.0026 | 0.0108 |
|  |  | VX | 0.0109 | 0.0024 | 0.0092 |
|  |  | XX | 0.0105 | 0.0022 | 0.0095 |
| Global Efficiency | Core | VA | 0.1914 | 0.0384 | 0.1710 |
|  |  | AX | 0.1840 | 0.0308 | 0.1236 |
|  |  | VX | 0.1835 | 0.0326 | 0.1273 |
|  |  | XX | 0.1785 | 0.0313 | 0.1183 |
|  | Auditory | VA | 0.1548 | 0.0284 | 0.1271 |
|  |  | AX | 0.1536 | 0.0244 | 0.1162 |
|  |  | VX | 0.1476 | 0.0292 | 0.1096 |
|  |  | XX | 0.1447 | 0.0359 | 0.1452 |
|  | Visual | VA | 0.1784 | 0.0378 | 0.1556 |
|  |  | AX | 0.1668 | 0.0381 | 0.1640 |
|  |  | VX | 0.1806 | 0.0364 | 0.1585 |
|  |  | XX | 0.1728 | 0.0303 | 0.1208 |
| Participation Coefficient | Core | VA | 0.3714 | 0.0431 | 0.1948 |
|  |  | AX | 0.3534 | 0.0700 | 0.2853 |
|  |  | VX | 0.3704 | 0.0558 | 0.2389 |
|  |  | XX | 0.3577 | 0.0754 | 0.3303 |
|  | Auditory | VA | 0.5476 | 0.0720 | 0.3150 |
|  |  | AX | 0.5379 | 0.0620 | 0.2309 |
|  |  | VX | 0.5488 | 0.0602 | 0.2090 |
|  |  | XX | 0.5283 | 0.0856 | 0.3155 |
|  | Visual | VA | 0.5298 | 0.0608 | 0.2724 |
|  |  | AX | 0.5067 | 0.0792 | 0.2809 |
|  |  | VX | 0.5379 | 0.0775 | 0.3507 |
|  |  | XX | 0.5309 | 0.0628 | 0.2422 |
| Path Length | Core | VA | 5.6181 | 1.2285 | 5.6601 |
|  |  | AX | 5.7470 | 1.0497 | 4.3581 |
|  |  | VX | 5.8214 | 1.1236 | 4.7610 |
|  |  | XX | 5.9817 | 1.2222 | 4.6854 |
|  | Auditory | VA | 6.8304 | 1.3604 | 6.6109 |
|  |  | AX | 6.8419 | 1.2709 | 6.5362 |
|  |  | VX | 7.1446 | 1.3467 | 4.8550 |
|  |  | XX | 7.4107 | 1.6014 | 6.3339 |
|  | Visual | VA | 5.9033 | 1.1644 | 4.7438 |
|  |  | AX | 6.3669 | 1.4215 | 6.1663 |
|  |  | VX | 5.8280 | 1.2433 | 5.9029 |
|  |  | XX | 6.0453 | 1.1623 | 5.1055 |
| Strength | Core | VA | 4.9518 | 1.4202 | 6.3766 |
|  |  | AX | 4.7618 | 1.2029 | 4.9113 |
|  |  | VX | 4.7455 | 1.2217 | 4.5728 |
|  |  | XX | 4.5594 | 1.0910 | 4.1179 |
|  | Auditory | VA | 3.2405 | 0.9266 | 4.2130 |
|  |  | AX | 3.2362 | 0.8057 | 3.9548 |
|  |  | VX | 2.9164 | 0.9660 | 3.7602 |
|  |  | XX | 2.8982 | 1.1518 | 5.0990 |
|  | Visual | VA | 4.3253 | 1.3138 | 5.2127 |
|  |  | AX | 3.9555 | 1.4528 | 5.8615 |
|  |  | VX | 4.5967 | 1.4946 | 5.8745 |
|  |  | XX | 4.3487 | 1.2512 | 4.5534 |
